# Anxiety in Mice: A Principal Component Analysis Study

**DOI:** 10.1155/2007/35457

**Published:** 2007-03-21

**Authors:** Yan Clément, Chantal Joubert, Caroline Kopp, Eve M. Lepicard, Patrice Venault, René Misslin, Martine Cadot, Georges Chapouthier

**Affiliations:** ^1^Groupe Hospitalier Pitié-Salpêtrière, CNRS UMR 7593, Université Paris 6, 91 Boulevard de l'Hôpital, 75634 Paris Cedex 13, France; ^2^Laboratoire de Biochimie Médicale et Biologie Moléculaire, CNRS UMR 6198, Université Reims Champagne-Ardenne, 51096 Reims Cedex, France; ^3^Laboratoire d'Ethologie et de Neurobiologie, URA CNRS 1295, Université Louis Pasteur, 7 Rue de l'Université, 67000 Strasbourg, France

## Abstract

Two principal component analyses of anxiety were undertaken investigating two strains of mice (ABP/Le and C57BL/6ByJ) in two different experiments, both classical tests for assessing anxiety in rodents. The elevated plus-maze and staircase were used for the first experiment, and a free exploratory paradigm and light-dark discrimination were used for the second. The components in the analyses produced definitions of four fundamental behavior patterns: novelty-induced anxiety, general activity, exploratory behavior, and decision making. We also noted that the anxious phenotype was determined by both strain and experimental procedure. The relationship between behavior patterns and the use of specific tests plus links with the genetic background are discussed.

## 1. INTRODUCTION

Most behavioral procedures for studying the pharmacology of anxiety use models involving nonconditioned behavioral responses that are usually based on novelty-induced variations in exploratory activity. Ethological observations show that while rodents naturally tend to explore a novel environment, open fields are aversive and counter normal behavioral responses [1–3]. The light-dark discrimination and elevated plus-maze tasks are used for the same purpose. In these tasks, pharmacological studies have shown that benzodiazepines (BZ) or 5-HT_1A_ agonists ligands have anxiolytic-like effects on mice, increasing time spent in the lit box and exploring the open arms in the elevated plus-maze [2, 4, 5], while BZ antagonists or inverse agonists and anxiogenic 5-HT drugs decreased both of these behavioral measurements [6–9]. In humans, two main types of anxiety which are well identified have been reported: “state” and “trait” anxieties [10]. “State anxiety” is anxiety that a subject experiences at a particular moment in time and which is increased in the presence of an anxiogenic stimulus. In contrast, “trait anxiety” does not vary from moment to moment and is considered to be an “enduring” feature in an individual [11–13]. In rodents, “state anxiety” has been extensively studied but “trait anxiety” is less well known. Belzung and Griebel proposed the light-dark task and the elevated plus-maze device as the most appropriate for assessing “state anxiety,” while the free-exploratory paradigm can be used for “trait anxiety” [4, 14]. Unlike most behavioral models using spontaneous aversion (unconditioned fear) to a new environment, the free-exploratory paradigm does not force the animal to explore. After 24-hour exposure to the two compartments (familiar/novel) of the apparatus, the animal can choose to explore familiar or novel areas. Thus, “trait anxiety” is associated with approach responses to the unfamiliar (novel) compartment being followed by avoidance reactions, while “state anxiety” is associated with neophobia to the new environment and/or avoidance reactions to an unprotected compartment when animals are forced to explore it.

To gain a better understanding as to whether specific behavioral variables can be related to “trait” or “state” anxiety, the aim of the present study was first to record behavioral patterns in four specific behavioral tests assessing “trait anxiety” (free-exploratory paradigm) and “state anxiety” (staircase, elevated plus-maze, and light-dark discrimination) in mice, and to carry out principal component analyses of the data, this being a commonly used method [15–21]. Second, many animal studies using inbred strains have reported strain differences in anxiety-related behavior, suggesting that genetic factors could be associated with anxious phenotypes [22–27]. We recently reported behavioral differences in the open-field and in the light-dark devices studying two inbred strains of mice: C57BL/6ByJ (B6) and ABP/Le (ABP), observing that ABP was anxious compared to B6 [28, 29]. B6 mice have often been used by scientists in behavioral and pharmacological studies, but there is insufficient knowledge of the ABP strain [30]. A study of anxiety-related behavior by principal component analysis was therefore undertaken on the two strains to provide a more accurate definition of the differential components and to test the hypothesis of genetic determinism for anxiety.

## 2. MATERIALS AND METHODS

### 2.1. Animals

The animals were reared in groups of 5 or 6 male and female mice from ABP/Le and C57BL/6ByJ parent strains bred in the laboratory in Paris. They were reared under standard conditions: temperature 23.5 ± 0.5°C, photoperiod 12 h/12 h with lights on between 8 am and 8 pm; food (IU UAR), tap water ad libitum, and dust-free sawdust bedding. The animals were given a two-week recovery period after being transported from Paris to Strasbourg.

### 2.2. Behavioral testing

At the beginning of the experiment, the animals were 10-week old ±2 weeks when tested and were test-naïve. They were first tested in the Paris laboratory, and two weeks later in the Strasbourg laboratory. In the first experiment (Paris), 94 mice were tested: 50 ABP mice (24 males and 26 females) and 44 B6/ByJ (29 males and 15 females). In the second experiment (Strasbourg), 81 mice (from a total of 94 sent to Strasbourg) were tested: 47 ABP (21 males and 26 females) and 34 B6/ByJ (24 males and 10 females). The experiments took place in a room outside the housing room between 1 pm and 5 pm. Data were recorded using a handheld computer (Psion Organiser). Animals were kept on a 12 h/12 h light/dark cycle with lights on at 1 am so that we could observe the animals under dim red light during their active period between 2 pm and 5 pm. There was a minimum interval of one week between experiments.

All experiments complied with the ethical guidelines laid down by the French Ministry of Agriculture and with the European Community Council Directive of November 24, 1986 (86/609/EEC).

## 3. EXPERIMENT 1

### 3.1. Elevated plus-maze

The apparatus was a polyvinylchloride plus-maze with two lit open arms (27×5 cm) and two closed arms (27×5×15 cm). The two closed arms were darkened with cardboard to block out the light. The arms radiated from a central platform (5×5 cm) [31]. The apparatus was mounted on a base which raised the arms to a height of 38.5 cm above the floor. To initiate the test session, the mouse was placed on the central platform, facing an open arm, and its behavior was videotaped for 5 minutes. The mouse was considered to be on the central platform whenever two paws were on it, and in one of the arms when all four paws were inside.

Parameters recorded were time spent on open arms (TOA) for anxiety-related behavior, the number of entries into open arms (OAE) and closed arms (CAE) for locomotor activity, the time spent in the central area (TCA) and stretched-attend posture (SAP) for avoidance behavior, and unprotected head dipping (HD) (i.e., the animal extending its head below the open arm) for exploration activity [32, 33].

### 3.2. Staircase

The device consisted of a white wooden staircase similar to the one used by Simiand et al. [34]. The staircase was enclosed between vertical walls and had 5 identical steps 2.5 cm high, 10 cm wide, and 7.5 cm deep. The height of the walls remained constant along the length of the staircase. Each mouse was placed individually at the bottom of the staircase for a 5-minute observation period. The number of steps climbed (STEPS) and the number of rearings (R) were recorded as anxiety indexes [35].

## 4. EXPERIMENT 2

### 4.1. Light-dark discrimination

The apparatus consisted of two polyvinylchloride boxes (20 × 20 × 14 cm) covered with Plexiglas [36]. One box was dark and covered with cardboard and the second box had a 100-watt bulb suspended 25 cm above it as the only source of light. An opaque tunnel (5 × 7 × 10 cm) ran between the two boxes. The apparatus was placed on a stand in the mouse room. The observer always sat in the same position, next to the apparatus. Each mouse was placed individually in the darkened box and recordings were made over a 5-minute period, counting the time spent in the lit box (TLB) and the number of transitions (TRANS) across the tunnel. A mouse with all four paws in the destination box was said to have made a transition.

### 4.2. Free-exploratory paradigm (Hughes Box)

The apparatus consisted of a polyvinylchloride box (30 × 20 × 20 cm) covered with Plexiglas and subdivided into six identical square exploration units, all interconnected by small doors [4]. A removable partition could be used to divide the apparatus in half lengthwise. Approximately 24 hours before testing, each subject was placed in one half of the apparatus, with the temporary partition in place, to be familiarized with it. The floor in this half was covered with sawdust and the animal was given unlimited access to food and water. The next day, the mouse was exposed to both the familiar and unfamiliar compartments when the temporary partition was removed, without removing the animal itself from the box. The subject was then observed under red light for 10 minutes. The parameters recorded were the number of units entered (locomotion) in the novel area (LOCN), the time spent in the novel side (TIME), the number of units entered in the familiar environment (LOCF), the number of rearings in the novel area (RN), the number of rearings in the familiar environment (RF), and the number of approach responses to the unfamiliar compartment followed by avoidance reactions (attempts, AT).

### 4.3. Component analysis

Principal component analysis and varimax rotation were conducted for each of the two experiments. An eigenvalue greater than 1 was set as the criterion for selecting components.

### 4.4. Statistical methods

The procedure used to compare the groups of mice was a multivariate analysis of variance with “strain” and “gender” as the main components, plus their interactions, followed by two-way ANOVAs for each component identified in the factorial analyses. Partial comparisons were done using the adjusted means. SAS was used for all the statistical analyses (factor and GLM).

## 5. RESULTS

### 5.1. Experiment 1 (N = 94)

The principal component analysis produced three factors with eigenvalues greater than 1. These three factors explain 67.9% of the variance in the correlation matrix and varimax rotation was performed on them. The rotated factor patterns are presented in Table 1. Calculations were made giving each mouse a score for each component.

*Component 1* (27.6% of variance) was mainly loaded by time spent in the center (TCA = 0.80), stretched-attend posture (SAP = 0.67), head dipping (HD = −0.75) and time spent in open arms (TOA = −0.45).

*Component 2* (21.6% of variance) was explained by steps climbed (STEPS = 0.91) and rearings (*R* = 0.62) in the staircase test.

*Component 3* (18.7% of variance) was loaded by the number of entries to open arms (OAE = 0.86) and closed arms (CAE = 0.83) in the elevated plus-maze.

MANOVA analysis of the scores for components 1, 2, and 3 from the principal component analysis, considered as dependent variables, showed significant effects for strain, gender, and Strain X Gender (*F*_(3,88)_ = 102.9, *P* < .0001; *F* = 4.31, *P* < .007; *F* = 8.4, *P* < .0001, resp.).

**Figure 1:**
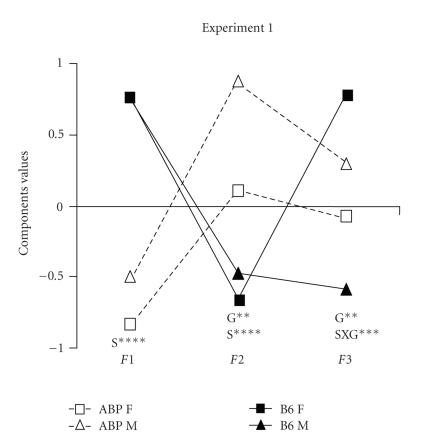
Mean scores by strain and gender of component values, S = strain effect; G = gender effect; SXG = strain-gender interaction, ** = *P* < .01; **** = *P* < .0001.

Profile analysis showed a level effect (Figure 1) for strain X gender (*F* = 13.3, *P* < .0002). The parallelism effect was significant for strain, gender, and strain X gender (Wilk's lambdas = 0.22, *P* < .0001; Λ = 0.87, *P* < .002; Λ = 0.90, *P* < .01, resp.).

ANOVA procedures revealed a strain effect for components 1 and 2 (*F*_(1,90)_ = 90.92, *P* < .0001; *F* = 36.54, *P* < .0001). Gender was significant for components 2 and 3 (*F* = 7.46, *P* < .008; *F* = 6.98, *P* < 0.01). Strain X gender was significant only for component 3 (*F* = 20.72, *P* < .0001).

### 5.2. Experiment 2 (N = 81)

The principal component analysis produced 4 components with eigenvalues greater than 1. These four components explain 76.9% of the variance in the correlation matrix and varimax rotation was performed on them. The rotated factor patterns are presented in Table 2. Calculations were made giving each mouse a score for each component.

*Component 1* explained 21.2% of variance. The number of locomotion events (LOCN = 0.82) and rearings (RN = 0.88) in the novel side mainly loaded this factor; time spent in the novel side (TIME = 0.45) also loaded the factor.

*Component 2* explained 19.0% of variance and was loaded by the number of avoidance reactions to unfamiliarity (AT = 0.83) and by time spent in the novel area (TIME = −0.70).

*Component 3* explained 18.8% of variance and was mainly loaded by rearings (RF = 0.62), locomotion in the familiar area (LOCF = 0.91), and time spent in the novel area (TIME = −0.41).

*Component 4* explained 17.9% of total variance and was loaded by the number of transitions (TRANS = 0.84) and time spent in the lit box of the light-dark apparatus (TLB = 0.81).

MANOVA analysis of the scores from the principal component analysis (components 1 to 4), considered as dependent variables, showed a significant strain effect (*F*_(4,74)_ = 9.38, *P* < .0001). The strain X gender effect was also significant (*F* = 4.03, *P* < 0.005).

**Figure 2:**
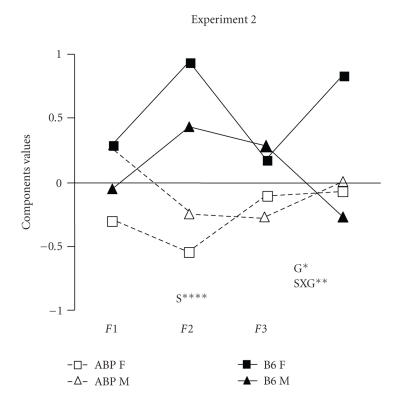
Mean scores by strain and gender of component values, S = strain effect; G = gender effect; SXG = strain-gender interaction, * = *P* < .04; ** = *P* < .01; **** = *P* < .0001.

A profile analysis (Figure 2) showed a level effect for strain (*F*_(1,77)_ = 22.10, *P* < .001) and for strain X gender (*F* = 9.87, *P* < .002). The parallelism effect was significant for strain (Wilk's lambda = 0.088, *P* < .02).

ANOVA procedures showed only a strain effect for component 2 (*F*_(1,77)_ = 28.19, *P* < .0001) and tended towards significance for component 3 (*P* < .06). Gender was significant for component 4 (*F* = 4.92, *P* < .03). Strain X gender was mainly significant for component 4 (*F* = 6.72, *P* < .01). For components 1 and 2, strain X gender tended towards significance, (*F* = 3.84, *P* < .06; *F* = 3.74, *P* < .06).

## 6. DISCUSSION

It is commonly known that rodents, when confronted with a novel environment, either explore it or try to escape; many behavioral procedures therefore use unconditioned responses to measure anxiety [30]. As several authors have proposed the distinction between “trait anxiety” and “state anxiety” [4, 13], a principal component analysis was performed on the data to set behavioral parameters related to each of the two forms of anxiety, and specifically to distinguish anxious responses from exploratory and locomotor activities. The elevated plus-maze, the light-dark choice procedure, and the staircase test were assumed to measure “state anxiety,” while the free-exploratory paradigm was used to assess “trait anxiety.”

Analyzing data from the staircase and elevated plus-maze procedures (experiment 1), a 3-component structure explained 70% of total variation. After rotation, time spent in the center (TCA) and stretched-attend posture (SAP) were positively loaded on component 1, while time in the open arms (TOA) and head dips (HD) negatively loaded on this factor. Component 2 was defined by rearings (R) and climbed steps (STEPS) in the staircase test. The number of entries into the open (OAE) and closed arms (CAE) of the plus-maze defined component 3.

In the second experiment, a four-component model explained approximately 80% of total variation for the data observed in the light-dark choice and in the free-exploratory paradigm. The variables contributing to components 1 to 3 were all recorded in the free-exploratory paradigm, while the variables of component 4 were all in the light-dark situation.

The factors that mainly loaded on components 1, 2, and 3 were, respectively, locomotion (LOCN) and rearings (RN) in the unfamiliar compartment, then the number of attempts (AT) and the time spent in the unfamiliar compartment (TIME), and last, locomotion (LOCF) and rearings (RF) in the familiar area. The number of transitions between the lit and dark boxes (TRANS) and the time spent in the lit box (TLB) defined component 4.

Overall, the component analyses suggest the following.

(1) The light-dark procedure and the staircase produce a different set of responses as behavioral variables measured in these procedures specifically loaded on their own component (component 4, experiment 2; and component 2, experiment 1). It may be deduced that TRANS and TLB in the light-dark task and STEPS and rearings in the staircase task can be considered as behavioral indexes that are independent from the other parameters.

(2) Since the number of entries into both open (OAE) and closed (CAE) arms of the plus-maze model coincided in the same component (component 3, experiment 1), these variables may be related to locomotion and may provide a general activity index.

(3) Exploratory behavior was estimated in different ways. It was noted that the exploratory response loaded on two separate components depending on whether the exploration was in familiar or unfamiliar compartments of the free-exploratory paradigm. LOCN and RN (component 1, experiment 2) expressed exploration in the novel area, while LOCF and RF (component 3, experiment 2) expressed exploration in the familiar area. Both correlated negatively to time spent in the unfamiliar environment (TIME).

(4) TCA and SAP, which were inversely associated with TOA and HD (component 1, experiment 1), may reflect “decision-making behavior” when deciding to enter the open arms of the plus-maze. The more time the animal spent in the centre, the less it explored the open arms. We hypothesized that avoidance to explore may indicate anxiogenic-like effects in the plus-maze paradigm, as with AT and TIME behavior parameters (component 2, experiment 2) in the free-exploratory paradigm. As these behavior patterns loaded on different components, they can be used to define different kinds of anxiety.

The time spent in the lit box and the number of transitions between the two boxes in the light-dark model, the time spent in the open arms in the elevated plus-maze, and the time spent in the novel side of the free exploration model are usually considered as a measurement of anxiety-related behavior: the more time an animal spends in the lit box and in the open arms, the less anxious it is [4, 30, 37]. Very few studies have reported data on the staircase test as a measurement of anxiety [34, 38, 39]. The authors of such papers, on rats, have suggested that the number of steps climbed may be a locomotor component index, and that rearings relate to anxiety. A recent ethopharmacological study reported an increase in both steps climbed and rearings by BALB/cBy mice given diazepam, suggesting that mice climbing the greatest number of steps and recording that the most rearings are less anxious [35].

Overall, our data tally with the literature and show that the number of transitions between lit and dark boxes is not linked to other locomotion variables, confirming that the parameter is not related to motor activation, but rather to a particular emotional state [2, 40, 41]. Although the light-dark choice situation measures “state anxiety,” our data suggest that the test also reveals a type of anxiety different from that measured by the plus-maze or the staircase procedures.

Previous plus-maze studies have suggested that open-arm entries and unprotected head dippings are the best indicators of anxiety. Total entries into closed and open arms were associated with locomotion, while total head dippings were associated with exploration, and the percentage of time spent in the center and stretched-attempt posture were associated with avoidance to explore [2, 32, 42]. Our results concur with the findings of these authors and confirm that exploration-related behaviors and locomotion loaded on separate components [31, 43]. Our study also suggests that exploration/novelty avoidance behavior can be a relevant index to measure anxiety. The time spent in open arms was a function of the time spent in the center, and the animals appeared to use the central area to “make decisions,” confirming the link between the central area and novelty avoidance. Finally, these data show the four behavioral procedures used in the study to be a means of identifying different responses for coping with novelty-induced anxiety. In the staircase and light-dark choice procedures, we can distinguish specific behavioral phenomena which may be defined as parameters for “state anxiety,” while general locomotion and exploration are defined in the plus-maze apparatus and the free-exploratory paradigm, respectively. Two other anxiety-related behavior patterns can be identified with these two procedures: “state anxiety” may be assessed through so-called “decision-making variables” in the plus-maze, and “trait anxiety” can be seen through “avoidance variables” in the free-exploratory paradigm. These data confirm previous studies showing that animal behavior recorded in these tests did not reflect the same emotional *status* [4, 11, 41, 44, 45]. The response patterns in both the free-exploration and plus-maze models offer potential for studying the effects of anxiogenic/anxiolytic drugs and could be included in pharmacological studies.

Many studies have pointed to great genetic variability in anxiety in different strains of mice [41, 46–49], suggesting that genetic background may modulate the biological processes involved in the physiopathology of disease etiology. We previously reported strain differences in the open-field and light-dark tests observing two strains of inbred mice, ABP/Le and C57BL/6ByJ: the ABP strain being described as more reactive than B6 [28, 29]. To further characterize and compare the behavior patterns of the two strains, and after a factorial analysis applied to data from the four experimental behavioral environments, we compared them, performing a profile analysis by a two-way ANOVA (Figures 1 and 2). We found a significant strain X gender interaction in both experiments for components 3 (experiment 1) and 4 (experiment 2), but since B6 females were different from all the others (*P* < .0001, for both experiments), the assumption was that the effect was only found with this population. The gender effect observed in components 3 (experiment 1) and 4 (experiment 2) may also be solely due to the female B6 group. However, the strain and gender effects observed in component 2 (experiment 1) specifically discriminated both strain and sex influences, and could be associated with differential behavioral patterns in the staircase test. Strain differences were also observed for components 1 (experiment 1) and 2 (experiment 2) and it was argued that they could be used to distinguish “state anxiety” from “trait anxiety.” Moreover, we noted differential profiles in strains for behavior and procedure (Figures 1 and 2). ABP was “higher” than B6 in the staircase test, but “lower” than B6 in the plus-maze and free-exploratory paradigm, suggesting different strain strategies in response to novelty.

To sum up, strain-related behavior patterns were found to be dependent on the behavioral situation and the genetic background. ABP strain could generally be described as more reactive than B6 in the staircase, and less reactive in both the free-exploration paradigm and the plus-maze test. The differences observed in “avoidance behavior” in free-exploration and “decision making” in the plus-maze models might reflect differential adaptive strategies when the animals are confronted with a conflict procedure, that is, having to choose between exploring a novel environment or staying in a protected area. The relationship therefore between these two behavioral profiles in the two experimental procedures could be further investigated by pharmacological and ethological studies with a view to gaining a better understanding of these behavioral “markers” for anxiety.

Many behavioral and pharmacological studies have used the B6 strain to measure anxiety and/or differential sensitivity to anxiolytic/anxiogenic drugs [50–53]. The B6 strain has been reported as not being “anxious” [48, 54, 55] and is more suitable for investigating the actions of anxiogenic drugs [36, 56, 57]. Very few authors have published data on ABP, the strain identified as being more “anxious” and more sensitive to convulsant drugs when compared to B6 [58, 59]. We can confirm that ABP mice explored less in the elevated plus-maze and more in the staircase device (experiment 1). They also recorded less “avoidance” behavior (experiment 2) than B6, suggesting that anxiogenic or anxiolytic *status* was dependent on the environment. The data are complex but tally with other data recorded in our and other laboratories and would suggest that the genetic basis for complex behavior is modulated by the genetic background, with the genotype being expressed in quite different ways according to the environment [60–63]. When testing drugs used to treat anxiety, the ABP strain may be more appropriate with experiments in the plus-maze, while B6 might be used in the staircase test for the same purpose. These variations also suggest that the anxious phenotype mainly depends on the interaction between genetic background and the experimental environment. It can be deduced that the choice of both the behavioral procedure and the strain is of crucial importance when testing anxiolytic and/or anxiogenic drugs. The present data could thus provide a useful guide for the pharmacological study of anxiety-related behavioral phenomena.

## 7. CONCLUSION

The present report is a principal component analysis study applied to two different genetic backgrounds and four behavioral paradigms known to evaluate novelty-induced anxiety in mice. We found that anxiety could be seen as four components: novelty-induced anxiety, general activity, exploratory behavior, and decision making. Of the different procedures available to assess anxiety-related behaviour, the staircase and light-dark test provide specific behavioral models for specific emotional states. Our data obtained studying two selected strains support the hypothesis that an anxious phenotype is mainly determined by the interaction between the genetic background and the experimental environment. The choice of the strain to investigate will depend on the environmental/experimental situation best suited to the requirements of the pharmacological study of anxiety-related behavior.

## Figures and Tables

**Table 1:** Rotated component patterns for experiment 1 (plus-maze and staircase). TOA = time spent in open arms; OAE = number of entries to open arms; CAE = number of entries to closed arms; TCA = time spent on the central area; SAP = stretched-attend posture; HD = unprotected head dipping (HD); steps = number of steps climbed; rearing = number of rearings. Only component patterns above 0.40 were recorded.

Variables	C 1	C 2	C 3

TOA	−0.45	—	—
OAE	—	—	0.86
CAE	—	—	0.83
TCA	0.80	—	—
SAP	0.67	—	—
HD	−0.75	—	—
Steps	—	0.91	—
Rearing	—	0.62	—

**Table 2:** Rotated component patterns for experiment 2 (light-dark discrimination and free-exploratory paradigm). TLB = time spent in lit box; Trans = number of transitions; LOCN = number of units entered (locomotion) in the novel area; time = time spent in the novel side; LOCF = number of units entered in the familiar environment; RN = number of rearings in the novel area; RF = the number of rearings in the familiar environment; AT = attempts, taht is, number of approach responses towards the unfamiliar compartment followed by avoidance reactions. Only component patterns above 0.40 were recorded.

Variables	C 1 21.2%	C 2 19.0%	C 3 18.8%	C 4 17.9%

TLB	—	—	—	0.81
Trans	—	—	—	0.84
LOCN	0.82	—	—	—
TIME	0.45	−0.70	−0.41	—
LOCF	—	—	0.91	—
RN	0.88	—	—	—
RF	—	—	0.62	—
AT	—	0.83	—	—
